# Surgical Management of a Giant Desmoid Fibromatosis of Abdominal Wall With Vessels Invasion in a Young Man: A Case Report and Review of the Literature

**DOI:** 10.3389/fsurg.2022.851164

**Published:** 2022-04-11

**Authors:** Jiming Zhao, Fajuan Cheng, Zhigang Yao, Bin Zheng, Zhihong Niu, Wei He

**Affiliations:** ^1^Department of Urology, Shandong Provincial Hospital Affiliated to Shandong First Medical University, Jinan, China; ^2^Department of Urology, Shandong Provincial Hospital Affiliated to Shandong University, Jinan, China; ^3^Department of Nephrology, Shandong Provincial Hospital Affiliated to Shandong First Medical University, Jinan, China; ^4^Department of Nephrology, Shandong Provincial Hospital Affiliated to Shandong University, Jinan, China; ^5^Department of Pathology, Shandong Provincial Hospital Affiliated to Shandong First Medical University, Jinan, China

**Keywords:** desmoid fibromatosis, abdominal, surgery, therapy, β-catenin

## Abstract

**Background:**

Desmoid fibromatosis (DF) is a rare clonal proliferation of fibroblasts and myofibroblasts. It develops in the connective tissues and does not metastasize but may infiltrate adjacent structures. Because of the rarity of these tumors and the unpredictable natural history of the disease, well-defined and precise guidelines of the optimal treatment for DF have not been formulated.

**Case Presentation:**

Here, we present a giant abdominal DF that invaded the right spermatic cord and iliac vessels. The lesion was excised with external iliac artery dissection; however, the vein was sacrificed. The abdominal wall defect was then repaired with a polypropylene mesh. The lesional cells are positive for β-catenin.

**Conclusions:**

In the past decades, there has been a change in the treatment of DF. The “wait and see” policy has been considered initially in most cases. Surgical intervention remains a valid option for symptomatic lesions. The optimal regimes of the tumor should not take the risk of making the patient more symptomatic than the lesion itself.

## Introduction

Desmoid fibromatosis (DF), also known as aggressive fibromatosis or deep fibromatosis, is a monoclonal fibroblastic and myofibroblastic proliferation, which arises from deep soft tissue and is characterized by infiltrative local progression but non-metastatic growth (1). The term “desmoid” is from the Greek word “desmos,” which means band or tendon, to illustrate the band- or tendon-like consistency of the tumor. DF is a relatively rare entity, making up just 0.03% of all neoplasms and 3% of all soft tissue tumors, with an annual incidence of 2–4 per million inhabitants in the European population and peak age of 30–40 years (2). It frequently occurs in the abdominal wall and the superficial muscular-aponeurotic tissue of extremities and is often divided into abdominal, intra-abdominal, and extra-abdominal types.

Approximately 15% of patients are related to familial adenomatous polyposis (FAP) (3). This disease is locally aggressive and has a high rate of recurrence, ranging from 18 to 56% (4, 5). The pathological diagnosis of DF is based on the uniform spindle-shaped cells with abundant collagen. About 75% of lesions are characteristic of positive nuclear β-catenin staining, but it is not disease-specific (6). Surgical excision is considered the standard treatment, but radiotherapy, medical therapy, or other systemic therapies are also employed. The influence of surgical margin, tumor size, and adjuvant treatment on the risk of local recurrence is disputed (7, 8).

The current study presents a case of a giant abdominal DF that invaded the right spermatic cord and iliac vessels. The clinical characteristics, surgical strategies, and prognosis are discussed, together with a literature review.

## Case Presentation

### Patient Information

**Figure 1:**
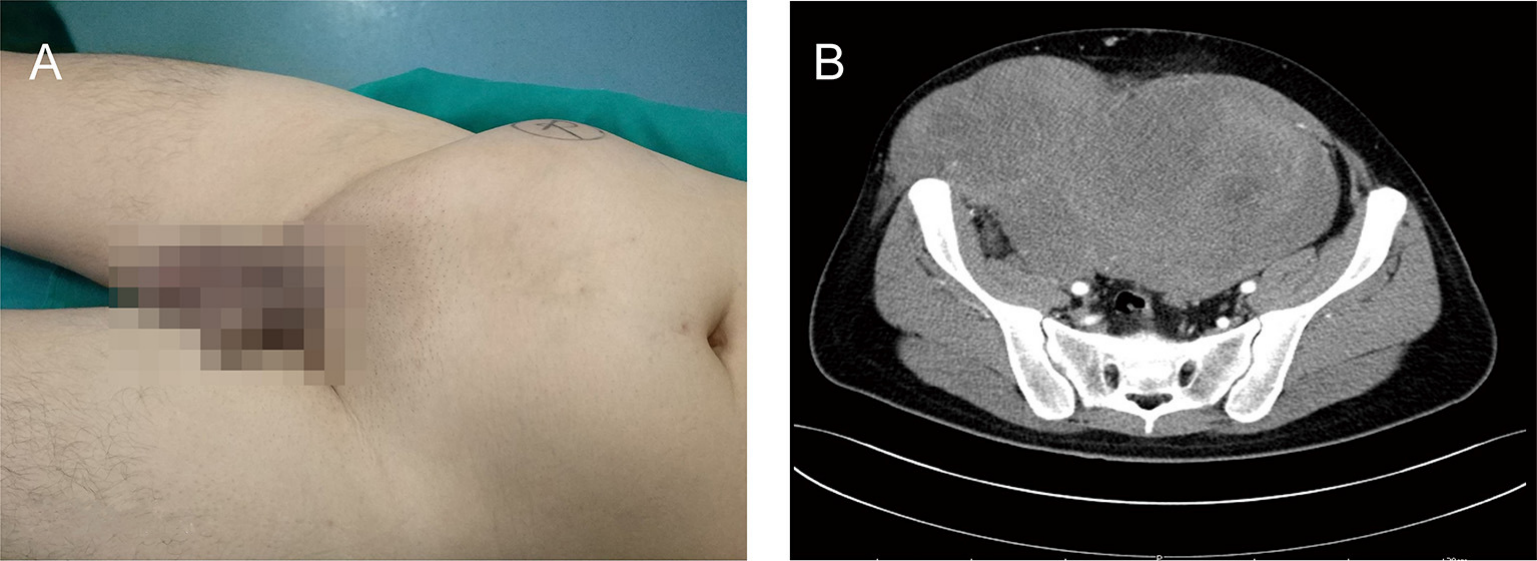
A clinical presentation of a giant right abdominal mass before surgical treatment. **(A)** The giant mass of right abdomen was visibly prominent. **(B)** The preoperative contrast-enhanced CT showed a large, inhomogeneous mass in the right abdominal wall that invaded right testis, spermatic cord, and right abdominal wall muscles, and demonstrated mild enhancement.

A 24-year-old male presented to our institution with a complaint of distention in the lower right abdominal wall for 1 year. He had no dizziness, fatigue, palpitation, hyperpyrexia, weight loss, or edema. He denied abdominal surgery and trauma. Physical examination revealed a large, muscle infiltrating prominent lump in the right abdomen, with an irregular shape, without varicosities, hydrocele, or varicocele (Figure 1A). The lesion was borderless and rubbery with hard consistency and was situated beneath the deep fascia, and was suspicious for muscle invasion. There was no rebound tenderness or guarding. All laboratory tests were within normal limits. The levels of tumor markers were also normal.

### Imaging Findings

The preoperative contrast-enhanced computed tomography (CT) was performed; it showed a large, inhomogeneous mass in the right abdominal wall that appeared to be continuous with the right testis, spermatic cord, and right abdominal wall muscles and demonstrated mild enhancement (Figure 1B). The lesion showed a spindle configuration with irregular margin, contacting with the abdominal muscular fascia forming the fascial tail sign. Bone involvement was present, located adjacent to the pubis and iliac crest. The nearby organs and tissues were compressed and pushed away.

### Therapeutic Interventions

**Figure 2:**
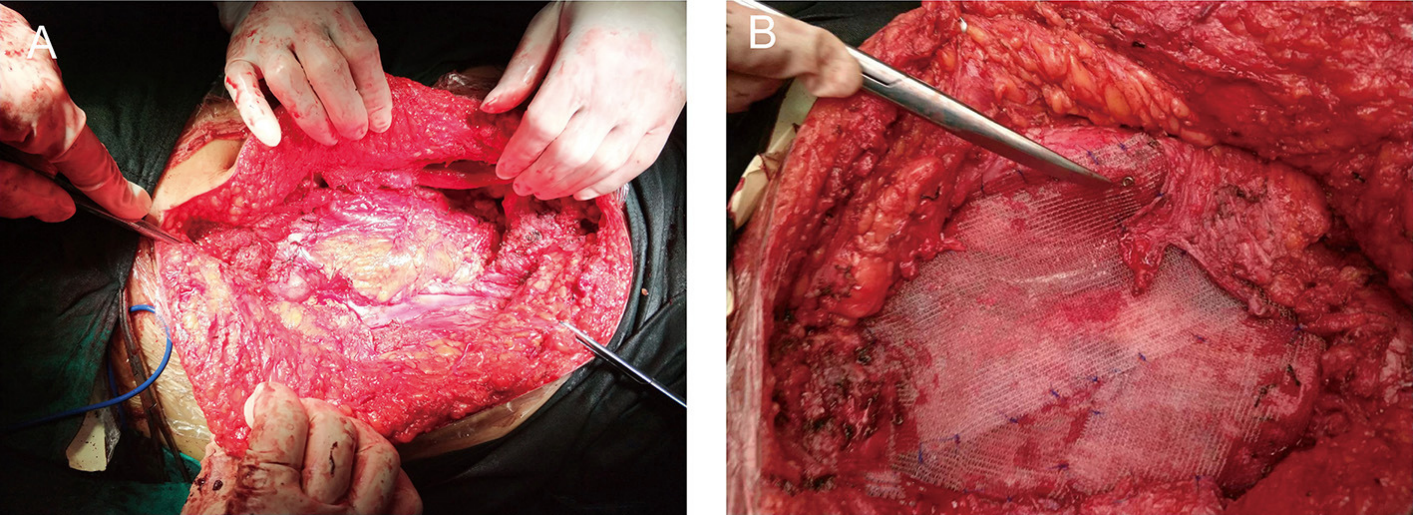
Images of the surgery. **(A)** Right abdominal wall defect created by the lumpectomy of the underlying musculature (right obliquus externus abdominis, obliquus internus abdominis, transverses abdominis, and rectus abdominis). **(B)** The defect was then repaired with a flat sheet of polypropylene mesh.

Upon completion of the preoperative workup, the patient was taken to the operating room. The lumpectomy was performed under general anesthesia. The patient underwent *en bloc* resection of the underlying musculature (right obliquus externus abdominis, obliquus internus abdominis, transverses abdominis, and rectus abdominis), right testis, and spermatic cord. It originated in the right aspect of the muscular fascia of the abdominal muscle. Indeed, adjacent structures were deformed and displaced because of the tumor's mass effect. The lesion was hard with an incomplete capsule and could not be separated from surrounding tissues easily. The tumor was excised with external iliac artery dissection; however, the vein was sacrificed because of tumor invasion. The abdominal wall defect (Figure 2A) was then repaired with a polypropylene mesh (Progrip™; Medtronic, Minneapolis, MN, USA) (Figure 2B). The right partial laparectomy site was then closed in a standard manner. The operating time was 6 h. The intraoperative estimated blood loss was 300 ml.

### Histopathological Findings

**Figure 3:**
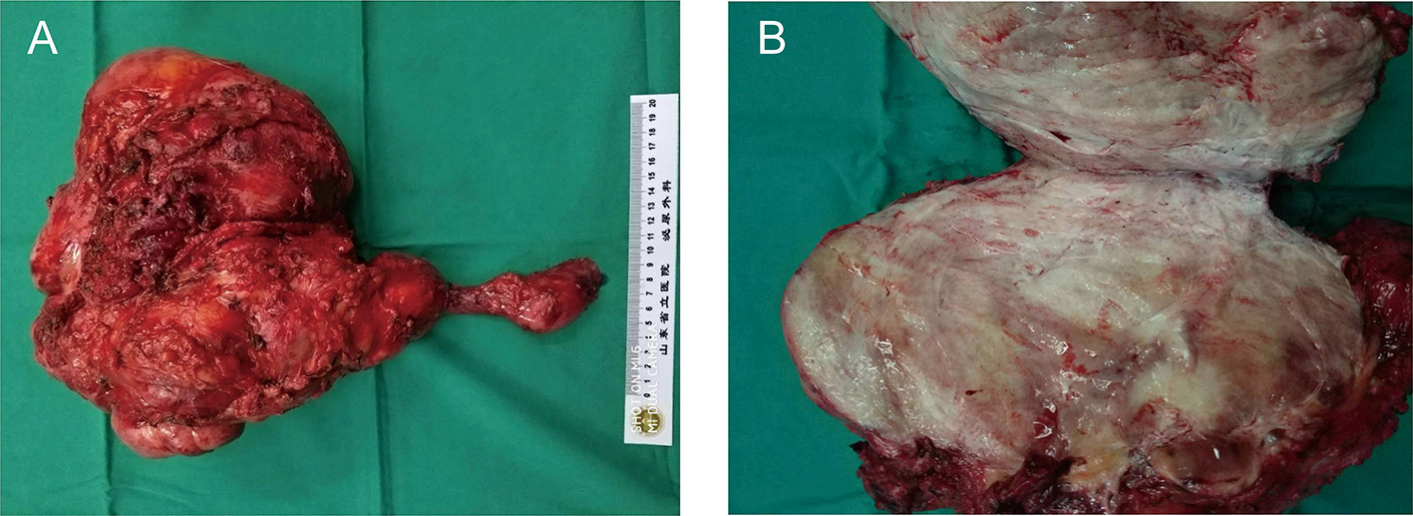
Surgical specimen of the right abdominal mass. **(A)** The surgical specimen of *en bloc* resection of the abdominal mass. **(B)** Cross-sectional view of the surgical specimen. The mass was large (30 cm × 24 cm × 15 cm in size), and the section was presented as fibrous tissue interleaving.

**Figure 4:**
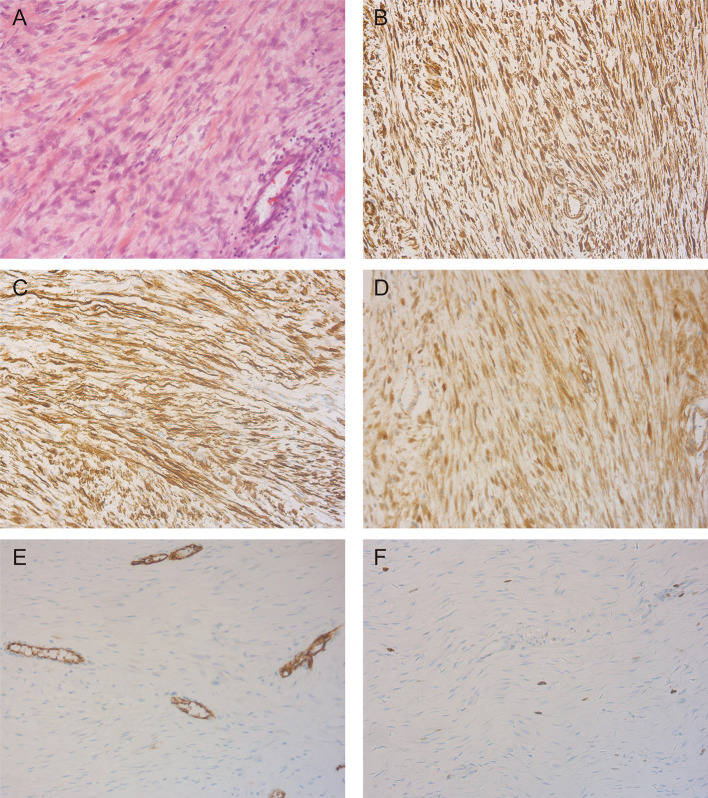
Representative photomicrographs in pathologic diagnosis. **(A)** The typical desmoid fibromatosis (DF) illustrating fascicles of spindle cells evenly arranged within a uniform collagenous stroma (H&E, X200). **(B)** Immunohistochemistry staining of vimentin showed a diffuse cytoplasmic staining pattern (X200). **(C)** Smooth muscle actin (SMA) positive expression is detected by immunohistochemistry staining (X200). **(D)** Immunoreactivity for β-catenin is seen in tumor cells (X200). **(E)** Staining patterns for CD34 (X200). **(F)** The tumor cells showed very low Ki-67 labeling index (X200).

This excised lesion was 30 cm × 24 cm × 15 cm in size, containing a grossly circumscribed firm texture (Figure 3A). On the cut surface, it was pale and had the appearance of fish meat (Figure 3B). The histological findings revealed a proliferation of spindle cells surrounded by abundant fibrillar collagen and occasional mitotic (Figure 4A). The lesion invasion of the underlying tissue involved resected muscles, soft tissue, and pubis. Immunohistochemical analyses were positive for vimentin (Figure 4B), smooth muscle actin (SMA) (Figure 4C), and β-catenin (Figure 4D), whereas CD34 was negative (Figure 4E). <8% of cells were Ki-67 positive (Figure 4F).

### Follow-Up and Outcomes

**Figure 5:**
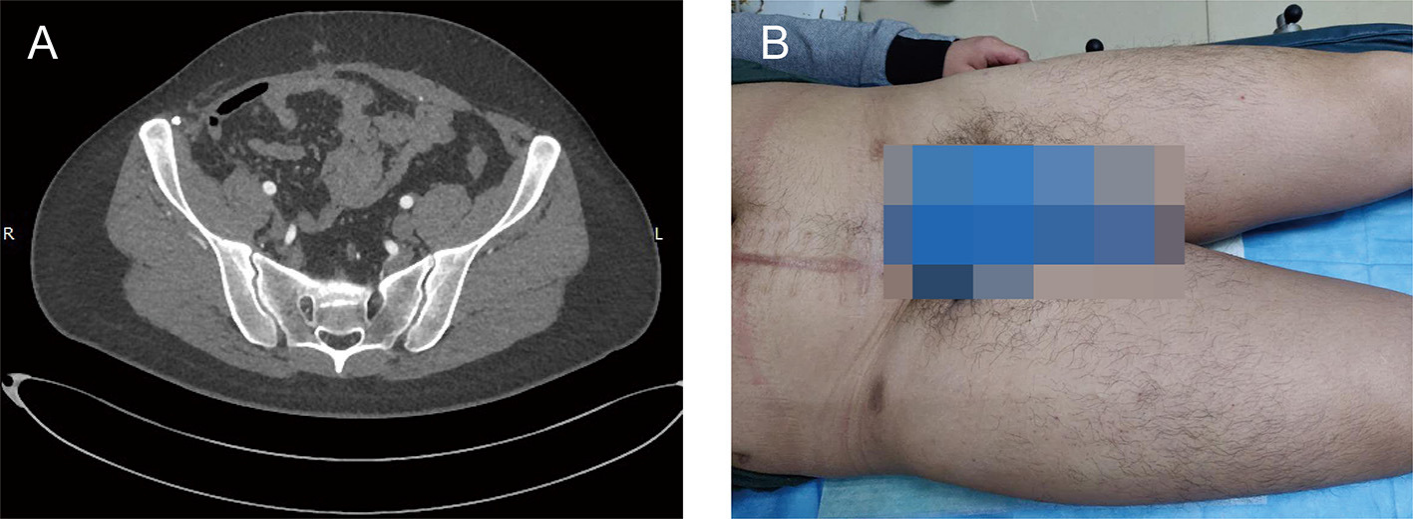
Clinical images at postoperative 20th month. **(A)** The postoperative contrast-enhanced CT showed no findings of recurrence. **(B)** Physical examination showed the swelling of right lower extremity.

Postoperative recovery was uneventful, and the patient was discharged on the 9th postoperative day. The patient remains in good health and shows no symptoms or imaging modality of recurrence at 20 months postoperative (Figure 5A). Physical examination revealed the swelling of the right lower extremity (Figure 5B).

## Discussion

Desmoid fibromatosis is a rare clonal proliferation of fibroblasts and myofibroblasts. This entity was classified as an intermediate (local aggressive) tumor by the WHO in the report on soft tissue tumor pathology and genetics in 2002 and divided into superficial and deep types (abdominal, extra-abdominal, and mesenteric) (9). It is a locally infiltrative collagen-forming tumor with potential for recurrence but not metastatic spread.

Desmoid fibromatosis develops in the connective tissues and does not metastasize but may infiltrate adjacent structures, extend along fascial planes, attach to and erode bones, and compress and engulf vessels, nerves, and other hollow organs (10). Although there are some reports of DF, it is rare for them to invade the spermatic cord. We did not encounter such entities in clinical practice. DF tends to occur in younger adults in the age of 20 and 30 years. The female:male ratio is 2:1. The lesion can commonly occur in women after pregnancy (11). The most common presentation of DF is a painless plaque or very firm lump with local pressure symptoms. The severe clinical problems could be caused by these lesions when the vital vessels, nerves, or abdominal organs are obstructed. Other complications include extremity ischemia, bowel fistulization, perforation, and bleeding.

The etiology of DF remains poorly understood. DF occurs rarely in the general population (2–4 per million people per year), but frequently in people with familial adenomatous polyposis (FAP) or Gardner's syndrome (12). FAP is a familial cancer predisposition condition which, if left untreated, results in colorectal carcinoma. About 2% of all DF arises in patients with FAP. These tumors are the result of mutations or changes in the *CTNNB1* gene and adenomatous polyposis coli (*APC*) gene (13, 14). *CTNNB1* gene mutations are associated with nearly 80% sporadic DF. *APC* gene mutations cause tumor in patients with FAP and 15% sporadic tumors. Antecedent trauma, surgical intervention, or repeated irritation may increase the risk of developing DF. The endocrine abnormality may also play a role in its development (15). In this present case, the patient and his parents have no history of FAP or Gardner's syndrome.

The standard imaging modalities for diagnosing DF are currently ultrasonography, magnetic resonance imaging (MRI), and CT (16–18). The ultrasound can be used with the palpable lesions of extremities, abdominal, chest wall, and breast. The sonographic appearance of DF is variable hyperechogenic areas within the tumor and well to poorly defined margins. The signal intensity of these lesions on MRI is variable. The typical appearance is similar to that of muscle on T1-weighted images and intermediate on T2-weighted images, which reflects the variable proportion and distribution of collagen and the cellularity of the tumor (19). The CT scan can also adequately evaluate the DF. On CT scans, this entity generally showed lower density with homogeneous attenuation and mild enhancement compared with muscle (20).

Desmoid fibromatosis is free of the capsule and sometimes circumscribed but more frequently invades surrounding tissues. The mass is composed of myofibroblasts, which have oval, pale staining, or vesicular nuclei, a variable proportion of cytoplasm, and ill-defined cell membranes. The lesion does not have a high mitotic rate, cellular atypia, vascular invasion, or necrosis (21). The lesion cells are positive for β-catenin and vimentin immunoreactivity and express SMA (22). This is very useful in the diagnosis of DF. The desmin is usually negative or occasionally focal positive staining. S-100, CD34, and CD117 are rarely expressed (23), which are useful in the differential diagnosis of leiomyoma, leiomyosarcoma, gastrointestinal stromal tumors, and other probable mesenchymal tumors (24).

Due to the rarity of these tumors and the unpredictable natural history of the disease, well-defined and precise guidelines of the optimal treatment for DF have not been formulated. Some lesions progressed quickly, but others ceased to grow and remained stable or even regressed without intervention. There is increasing literature supporting active surveillance as a primary strategy for asymptomatic patients with lesions in non-life-threatening locations (12, 25, 26). The initial progression is followed by regression in about half of patients (1). In the other half of patients, continuous progression or worsening mandates active interventions, which are utilized based on the tumor location, complication, and patient general status. Therefore, for patients with mild symptoms or mild progression, the “wait and see” policy of a duration of 12–24 months may be appropriate.

The surgical intervention is the preferred approach to remove the tumor, especially for patients with severe symptoms, serious complications, rapid progression, and important organ involvement (27). The complete excision is recommended to ensure negative margins (28). However, it is controversial whether the positive surgical margin affects recurrence. A multidisciplinary center retrospective study analyzed and demonstrated that an infiltrated margin had no significant effect on the prognosis of patients (7). In contrast, another study reported that an invasion of major vessels and nerves and surgical margin was risk factor for postoperative recurrence (29). Although positive margins are associated with a higher recurrence rate, it does not affect the overall survival. Therefore, the goal of surgical techniques is wide excision with the preservation of form and function and ensuring the survival benefit (30). The risk of local recurrence depends on many different factors, such as sex, age, location, size, multifocality, and genetic alterations (7). The abdominal lesions are associated with a better prognosis, followed by intra-abdominal. The extra-abdominal lesions have a higher potential of progression (31). For large abdominal defects, a surgical reconstruction may be repaired by direct sutures or by using synthetic meshes or myocutaneous flaps. In the present case, the peritoneal defect was replaced with a polypropylene mesh.

For the postoperative recurring lesions, those not susceptible to complete surgical intervention, or contraindication for systemic therapy, local irradiation is recommended (32). However, intra-abdominal tumors are not candidates for radiotherapy because of the side effects. Systemic therapy may be a valuable option in treating patients with DF who are unable to receive surgery, with positive surgical margins after surgery, or with postoperative recurrence (32). Systemic therapy comprises anti-estrogenic drugs (toremifene and tamoxifen), non-steroidal anti-inflammatory agents (celecoxib, meloxicam, sulindac, and indomethacin), cytotoxic chemotherapy (doxorubicin, methotrexate, and vinblastine), and target therapy (sorafenib, sunitinib, and imatinib). The anti-estrogenic drugs may be applied alone or in combination with non-steroidal anti-inflammatory drugs (NSAIDs) as an initial medical treatment for the low costs, rare adverse effects, and limited toxicity (33, 34). As the response rate is low and the relationship between regression is unclear, the anti-tumor effect of these medical reagents may have been overemphasized in reality (32). A low-dose cytotoxic regimen with methotrexate and vinblastine/vinorelbine has a favorable side effect profile and no late adverse effects or risks, which is employed in children and patients with few symptoms (35, 36). The conventional-dose chemotherapy using anthracycline-based regimens is used if a more rapid response is desired (37). Tyrosine kinase inhibitors, such as sorafenib, sunitinib, and imatinib have been evaluated in the management of DF. Imatinib showed a limited response in three phase 2 trials (38). In a phase-3 randomized placebo-controlled trial, the therapy with sorafenib appeared to be effective in slowing the progression in patients with DF (39). The response rate was more than 30%, with almost all patients having responded and without progression. The sorafenib is an important reagent in the armamentarium to treat DF and was selected for patients with recurrent, progressive lesions.

Desmoid fibromatosis is a rare clonal proliferation of fibroblasts and myofibroblasts. It occurs more frequently in the abdominal wall and extremities but seldom arises from the genitourinary system. To the best of our knowledge, this is the first report of a giant DF of abdominal wall with spermatic cord, and iliac vessel invasion received surgical intervention. In our case, we performed complete surgical resection. The surgical margin is negative. As the role of adjuvant therapy (such as, chemotherapy, hormonal therapy, and tyrosine kinase inhibitors) requires elucidation, we did not conduct additional therapeutic interventions except for routine follow-up. The imaging examination was utilized every 6 months for 3 years. Although there has been no recurrence for 3 years and 6 months in this case, long follow-up remains necessary.

## Conclusion

In conclusion, DF is a rare, locally aggressive proliferative mesenchymal tumor with a high incidence of recurrence. In the past decades, there has been a change in the treatment of DF. The “wait and see” policy has been considered initially in most cases. Surgical intervention remains a valid option for symptomatic lesions. The optimal regimes of the tumor should not take the risk of making the patient more symptomatic than the lesion itself. The present case report and literature review may be helpful to improve our knowledge of the disease and in the diagnosis and treatment of DF.

## Data Availability Statement

The original contributions presented in the study are included in the article/supplementary material, further inquiries can be directed to the corresponding author.

## Ethics Statement

The case report was approved by the Institutional Review Board of the Ethics Committee of Shandong Provincial Hospital (NO. SWYX2020-063). Informed written consent was obtained from the patient for publication.

## Author Contributions

WH and FC: conception and design. WH: administrative support. JZ, FC, and ZY: provision of study materials or patients. WH, ZY, and ZN: collection and assembly of data. WH, JZ, FC, BZ, and ZN: data analysis and interpretation. All authors: manuscript writing and final approval of manuscript. All authors contributed to the article and approved the submitted version.

## Funding

This work was supported by a grant from the FC from Shandong Provincial Natural Science Foundation (Grant No. ZR2016HB09) and the National Natural Science Foundation of China (Grant No. 81601421).

## Conflict of Interest

The authors declare that the research was conducted in the absence of any commercial or financial relationships that could be construed as a potential conflict of interest.

## Publisher's Note

All claims expressed in this article are solely those of the authors and do not necessarily represent those of their affiliated organizations, or those of the publisher, the editors and the reviewers. Any product that may be evaluated in this article, or claim that may be made by its manufacturer, is not guaranteed or endorsed by the publisher.

## Abbreviations

DF: desmoid fibromatosis
FAP: familial adenomatous polyposis
CT: computed tomography
IHC: Immunohistochemistry
SMA: smooth muscle actin
APC: adenomatous polyposis coli
MRI: magnetic resonance imaging
NSAIDs: non-steroidal anti-inflammatory drugs.

## References

[1] Kasper B Baumgarten C Garcia J Bonvalot S Haas R Haller F. An update on the management of sporadic desmoid-type fibromatosis: a European consensus initiative between sarcoma PAtients EuroNet (SPAEN) and European organization for research and treatment of cancer (EORTC)/soft tissue and bone sarcoma group (STBSG). Ann Oncol. (2017) 28:2399–408. 10.1093/annonc/mdx323 28961825 PMC5834048

[2] Penel N Coindre J-M Bonvalot S Italiano A Neuville A Le Cesne A. Management of desmoid tumours: a nationwide survey of labelled reference centre networks in France. Eur J Cancer. (2016) 58:90–6. 10.1016/j.ejca.2016.02.008 26974708

[3] Schiessling S Kihm M Ganschow P Kadmon G Büchler MW Kadmon M. Desmoid tumour biology in patients with familial adenomatous polyposis coli: desmoid tumour biology in familial adenomatous polyposis. Br J Surg. (2013) 100:694–703. 10.1002/bjs.9053 23334997

[4] Bonvalot S Eldweny H Haddad V Rimareix F Missenard G Oberlin O. Extra-abdominal primary fibromatosis: aggressive management could be avoided in a subgroup of patients. Eur J Surg Oncol. (2008) 34:462–8. 10.1016/j.ejso.2007.06.006 17709227

[5] van Broekhoven DLM Verhoef C Elias SG Witkamp AJ van Gorp JMHH van Geel BAN. Local recurrence after surgery for primary extra-abdominal desmoid-type fibromatosis: recurrence of desmoid-type fibromatosis. Br J Surg. (2013) 100:1214–9. 10.1002/bjs.9194 23804156

[6] Bhattacharya B Dilworth HP Iacobuzio-Donahue C Ricci F Weber K Furlong MA. Nuclear b-catenin expression distinguishes deep fibromatosis from other benign and malignant fibroblastic and myofibroblastic lesions. Am J Surg Pathol. (2005) 29:653–9. 10.1097/01.pas.0000157938.95785.da 15832090

[7] Salas S Dufresne A Bui B Blay J-Y Terrier P Ranchere-Vince D. Prognostic factors influencing progression-free survival determined from a series of sporadic desmoid tumors: a wait-and-see policy according to tumor presentation. J Clin Oncol. (2011) 29:3553–8. 10.1200/JCO.2010.33.5489 21844500

[8] Nuyttens JJ Rust PF Thomas CR Turrisi AT. Surgery versus radiation therapy for patients with aggressive fibromatosis or desmoid tumors: a comparative review of 22 articles. Cancer. (2000) 88:1517–23. 10.1002/(SICI)1097-0142(20000401)88:7<1517::AID-CNCR3>3.0.CO;2-9 10738207

[9] Fletcher CD. WHO Classification of Tumor. Pathology and Genetics of Tumor of Soft Tissue and Bone. 3rd ed. Lyon: IARC Press (2002).

[10] Kasper B Ströbel P Hohenberger P. Desmoid tumors: clinical features and treatment options for advanced disease. Oncologist. (2011) 16:682. 10.1634/theoncologist.2010-0281 21478276 PMC3228186

[11] Gounder MM Lefkowitz RA Keohan ML D'Adamo DR Hameed M Antonescu CR. Activity of Sorafenib against desmoid tumor/deep fibromatosis. Clin Cancer Res. (2011) 17:4082–90. 10.1158/1078-0432.CCR-10-3322 21447727 PMC3152981

[12] Eastley N McCulloch T Esler C Hennig I Fairbairn J Gronchi A. Extra-abdominal desmoid fibromatosis: a review of management, current guidance and unanswered questions. Eur J Surg Oncol. (2016) 42:1071–83. 10.1016/j.ejso.2016.02.012 26965303

[13] Colombo C Miceli R Lazar AJ Perrone F Pollock RE Le Cesne A. CTNNB1 45F mutation is a molecular prognosticator of increased postoperative primary desmoid tumor recurrence: an independent, multicenter validation study. Cancer. (2013) 119:3696–702. 10.1002/cncr.28271 23913621

[14] Sinha A Tekkis PP Gibbons DC Phillips RK Clark SK. Risk factors predicting desmoid occurrence in patients with familial adenomatous polyposis: a meta-analysis. Colorectal Dis. (2011) 13:1222–9. 10.1111/j.1463-1318.2010.02345.x 20528895

[15] Gansar GF Markowitz IP Cerise EJ. Thirty years of experience with desmoid tumors at charity hospital. Am Surg. (1987) 53:318–9. 3579044

[16] Casillas J Sais GJ Greve JL Iparraguirre MC Morillo G. Imaging of intra- and extra-abdominal desmoid tumors. Radiographics. (1991) 11:959–68. 10.1148/radiographics.11.6.1749859 1749859

[17] Dinauer PA Brixey CJ Moncur JT Fanburg-Smith JC Murphey MD. Pathologic and MR imaging features of benign fibrous soft-tissue tumors in adults. Radiographics. (2007) 27:173–87. 10.1148/rg.271065065 17235006

[18] Einstein DM Tagliabue JR Desai RK. Abdominal desmoids: CT findings in 25 patients. AJR Am J Roentgenol. (1991) 157:275–9. 10.2214/ajr.157.2.1853806 1853806

[19] Quinn SF Erickson SJ Dee PM Walling A Hackbarth DA Knudson GJ. Imaging in fibromatosis: results in 26 patients with pathologic correlation. AJR Am J Roentgenol. (1991) 156:539–42. 10.2214/ajr.156.3.1899752 1899752

[20] Xu H Koo HJ Lim S Lee JW Lee HN Kim DK. Desmoid-type fibromatosis of the thorax: CT, MRI, and FDG PET characteristics in a large series from a tertiary referral center. Medicine (Baltimore). (2015) 94:e1547. 10.1097/MD.0000000000001547 26402812 PMC4635752

[21] Kasper B Baumgarten C Bonvalot S Haas R Haller F Hohenberger P. Management of sporadic desmoid-type fibromatosis: a european consensus approach based on patients' and professionals' expertise – a Sarcoma patients EuroNet and european organisation for research and treatment of cancer/soft tissue and bone sarcoma group initiative. Eur J Cancer. (2015) 51:127–36. 10.1016/j.ejca.2014.11.005 25434922

[22] Ng TL Gown AM Barry TS Cheang MCU Chan AKW Turbin DA. Nuclear beta-catenin in mesenchymal tumors. Mod Pathol. (2005) 18:68–74. 10.1038/modpathol.3800272 15375433

[23] Leithner A Gapp M Radl R Pascher A Krippl P Leithner K. Immunohistochemical analysis of desmoid tumours. J Clin Pathol. (2005) 58:1152–6. 10.1136/jcp.2005.026278 16254103 PMC1770757

[24] Lacka DE Nasierowska-Guttmejer A. Fibromatosis – immunohistochemical evaluation, differential diagnosis from gastrointestinal tumors, and other mesenchymal tumours. (2019) 14:79–85. 10.5114/pg.2019.83429 30944681 PMC6444105

[25] Bonvalot S Ternès N Fiore M Bitsakou G Colombo C Honoré C. Spontaneous regression of primary abdominal wall desmoid tumors: more common than previously thought. Ann Surg Oncol. (2013) 20:4096–102. 10.1245/s10434-013-3197-x 24052312

[26] ESMO/European Sarcoma Network Working Group. Soft tissue and visceral sarcomas: ESMO clinical practice guidelines for diagnosis, treatment and follow-up. Ann Oncol. (2014) 25(Suppl 3):iii102–12. 10.1093/annonc/mdu254 25210080

[27] Gounder MM Thomas DM Tap WD. Locally aggressive connective tissue tumors. J Clin Oncol. (2018) 36:202–9. 10.1200/JCO.2017.75.8482 29220303 PMC6804876

[28] Escobar C Munker R Thomas JO Li BD Burton GV. Update on desmoid tumors. Ann Oncol. (2012) 23:562–9. 10.1093/annonc/mdr386 21859899

[29] Wang Y Guo W Sun K Yang R Tang X Ji T. Postoperative recurrence of desmoid tumors: clinical and pathological perspectives. World J Surg Oncol. (2015) 13:26. 10.1186/s12957-015-0450-8 25888954 PMC4329213

[30] Sharma A Ngan B-Y Sándor GKB Campisi P Forte V. Pediatric aggressive fibromatosis of the head and neck: a 20-year retrospective review. J Pediatr Surg. (2008) 43:1596–604. 10.1016/j.jpedsurg.2008.02.001 18778992

[31] Mullen JT Delaney TF Kobayashi WK Szymonifka J Yeap BY Chen Y-L. Desmoid tumor: analysis of prognostic factors and outcomes in a surgical series. Ann Surg Oncol. (2012) 19:4028–35. 10.1245/s10434-012-2638-2 22965569

[32] Gronchi A Jones RL. Treatment of desmoid tumors in 2019. JAMA Oncol. (2019) 5:567–8. 10.1001/jamaoncol.2018.6449 30703188

[33] Fiore M Colombo C Radaelli S Callegaro D Palassini E Barisella M. Hormonal manipulation with toremifene in sporadic desmoid-type fibromatosis. Eur J Cancer. (2015) 51:2800–7. 10.1016/j.ejca.2015.08.026 26602014

[34] Quast DR Schneider R Burdzik E Hoppe S Möslein G. Long-term outcome of sporadic and FAP-associated desmoid tumors treated with high-dose selective estrogen receptor modulators and sulindac: a single-center long-term observational study in 134 patients. Fam Cancer. (2016) 15:31–40. 10.1007/s10689-015-9830-z 26275868

[35] Skapek SX Ferguson WS Granowetter L Devidas M Perez-Atayde AR Dehner LP. Vinblastine and methotrexate for desmoid fibromatosis in children: results of a pediatric oncology group phase II trial. J Clin Oncol. (2007) 25:501–6. 10.1200/JCO.2006.08.2966 17290057

[36] Palassini E Frezza AM Mariani L Lalli L Colombo C Fiore M. Long-term efficacy of methotrexate plus vinblastine/vinorelbine in a large series of patients affected by desmoid-type fibromatosis. Cancer J. (2017) 23:86–91. 10.1097/PPO.0000000000000254 28410293

[37] Garbay D Le Cesne A Penel N Chevreau C Marec-Berard P Blay J-Y. Chemotherapy in patients with desmoid tumors: a study from the French sarcoma group (FSG). Ann Oncol. (2012) 23:182–6. 10.1093/annonc/mdr051 21444357

[38] Penel N Le Cesne A Bonvalot S Giraud A Bompas E Rios M. Surgical versus non-surgical approach in primary desmoid-type fibromatosis patients: a nationwide prospective cohort from the French sarcoma group. Eur J Cancer. (2017) 83:125–31. 10.1016/j.ejca.2017.06.017 28735069

[39] Gounder MM Mahoney MR Van Tine BA Ravi V Attia S Deshpande HA. Sorafenib for advanced and refractory desmoid tumors. N Engl J Med. (2018) 379:2417–28. 10.1056/NEJMoa1805052 30575484 PMC6447029

